# The Rare Occurrence of Giant Cell Tumor of the Proximal Tibia With Pathological Fracture in an Elderly Male: A Case Report

**DOI:** 10.7759/cureus.43102

**Published:** 2023-08-07

**Authors:** Madhavi M Kandarkar, Shivshankar Jadhav, Sanket M Kandarkar, Shubham Vaidya

**Affiliations:** 1 Department of Musculoskeletal Physiotherapy, Datta Meghe Institute of Medical Sciences, Wardha, IND; 2 Department of Orthopedic Surgery, Datta Meghe Institute of Medical Sciences, Wardha, IND; 3 Department of Orthopedic Surgery, Pravara Institute of Medical Sciences, Ahmednagar, IND; 4 Department of Orthopedic Surgery, Lata Mangeshkar Hospital, Nagpur, IND

**Keywords:** giant cell tumor of the bone, cementation, curettage, proximal tibia fracture, excisional biopsy

## Abstract

Giant cell tumor of the bone (GCTB) is a benign bone tumor that can occasionally progress to malignancy, usually in chronic cases. It is a common benign and aggressive bone tumor that affects patients aged between 20 and 45 years. The most common location is the knee joint. It manifests as a painless or occasionally painful swelling over the affected area. A case of giant cell tumor (GCT) of the proximal tibia in a 72-year-old male is reported here, which was difficult to diagnose as it is rarely found in the geriatric age group. The patient came with a chief complaint of pain and swelling over his left knee for two months with a history of trauma to the knee a couple of times. On clinical examination, the patient had grade 3 tenderness and swelling on the anterolateral aspect of the knee extending toward the proximal tibia. The swelling was well-defined, smooth, firm, and uniform in consistency with dimensions of 15 cm × 12 cm. The swelling was moveable sideways, and the movement of the knee suggested that it was not attached to the underlying bone. As per the age and history of the rapid-growing lesion, we suspected malignancy, but clinical findings were pointing toward benign tumor. X-ray of the affected knee was done, which revealed a soft tissue mass with the involvement of the bone. Magnetic resonance imaging (MRI) of the knee revealed a soft tissue mass with the cortical breach. An open biopsy was done for the confirmation of the diagnosis, which was later reported and confirmed as a giant cell tumor of the proximal tibia. As bone tumor is associated with a cortical breach and pathological fracture, it was classified under Campanacci grade 3, for which an en bloc resection and open reduction and internal fixation with plate osteosynthesis with bone cementing and bone grafting were done followed by knee bending physiotherapy and gradual weight-bearing. Finally, the knee function was improved with pain relief.

## Introduction

Although giant cell bone tumors are typically benign, they can occasionally be malignant and locally aggressive [1,2]. Bone giant cell tumor (GCT) was first described by Sir Astley Cooper in 1818. GCT is more prevalent in skeletally mature people, peaking in the 20-40 age range, with females slightly outnumbering males. Patients with open epiphyses account for less than 2% of cases, and patients 65 years old and older account for less than 10% [3-5]. It accounts for around 5% of primary bone tumors and 20% of benign bone tumors. Despite being a benign skeletal tumor, giant cell tumor of the bone (GCTB) is notorious for its aggressive local behavior and high rate of recurrence following curettage combined with adjuvant therapy (such as the additional removal of debris with a high-speed burr, cryotherapy using liquid nitrogen, chemical debridement using phenol, or bone cementing) [6-9]. For 10 weeks, the patient went to physiotherapy five days per week. The sessions comprised joint mobilization, stretching, sensory reeducation methods, electrotherapeutic modalities, thermotherapy, and range of motion (ROM) exercises [10]. The epiphysial-metaphyseal region of the long bones is the most common site for GCTB (70%-90%); the majority of this lesion extends within 1 cm of the affected bone's subarticular region. The presence of underlying trauma necessitates the treatment of the tumor, or it may result in pathological fracture. A tumor that has entered the subarticular space causes a pathological fracture. GCTB is most commonly found in the distal femur, followed by the proximal tibia, distal radius, sacrum, and proximal humerus [11]. Atypical GCT sites include the hands, feet, patella, and talus; atypical sites are common in multicentric GCT. Unusual GCT locations include the vertebral bodies and the mobile spine's posterior components. Physical therapy has been demonstrated to enhance patient performance, as well as the quality of life in postsurgical scenarios since a complete range of motion at the level of the knee joint cannot be regained following final surgery. The patient's primary worries were discomfort and edema in the wrists, as well as a decline in strength, power, and range of motion. X-ray, histopathology, and magnetic resonance imaging (MRI) all revealed a giant cell tumor. The velocity of nerve conduction confirms nerve injury.

## Case presentation

**Figure 1:**
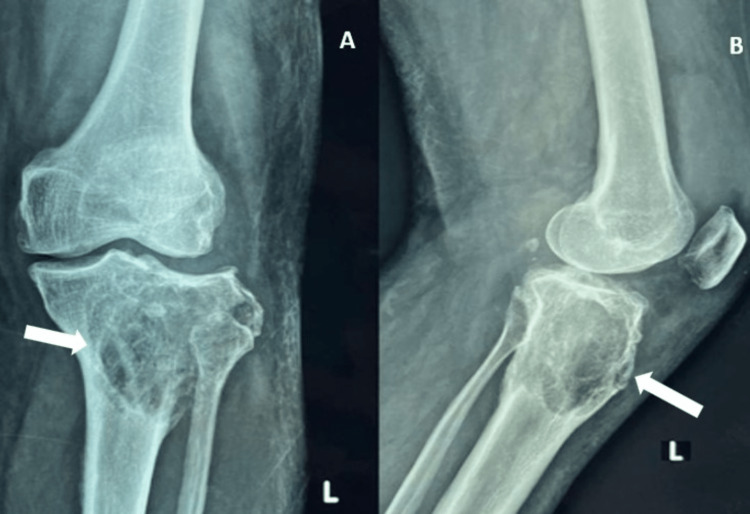
Anteroposterior (AP) and lateral view X-rays of the left knee showing osteolytic lesion involving the proximal tibia A and B are anteroposterior and lateral views of the left knee, showing bony swelling with a soap bubble appearance over the proximal third aspect of the tibia

**Figure 2:**
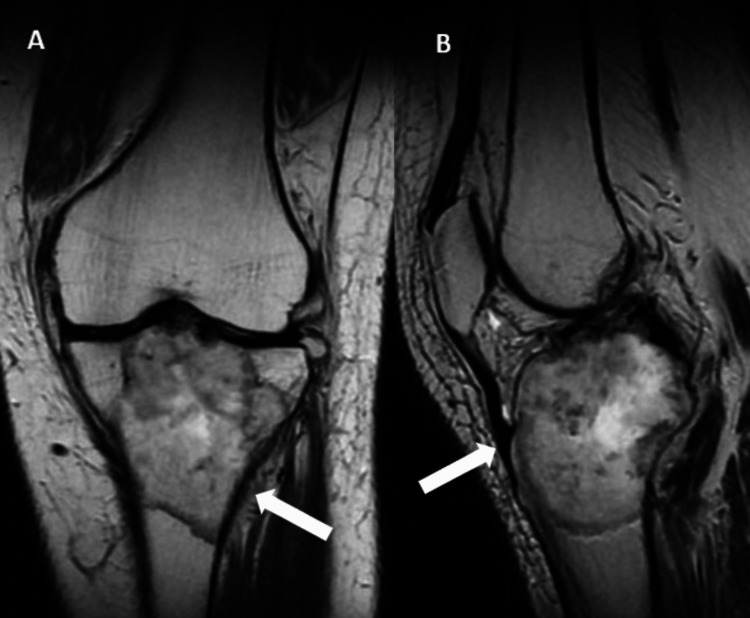
MRI scan of the left knee A and B show cortical breach and soft tissue shadow in the proximal tibia MRI: magnetic resonance imaging

A 72-year-old male obese patient with a history of systemic hypertension, who is a farmer by occupation, visited the orthopedics department with chief complaints of pain and swelling over the left knee for two months with a history of falls for two times in the last 20 days and an inability to bear weight since the last 20 days. The pain was associated with swelling, which was insidious in onset and gradually progressive in nature. The pain and swelling occurred without any prior trauma. The swelling grew in size over time, and the pain got worse when standing or walking. The pain aggravated after the fall, and the patient was unable to bear weight. The pain interferes with the patient's daily activities. The patient was advised to do some investigations such as X-rays (Figure 1), MRI (Figure 2), and Doppler and was diagnosed with a giant cell tumor and got operated for the same. The patient was referred for physiotherapy on postoperative day 2, and since then, the physiotherapy session has been going on.

**Figure 3:**
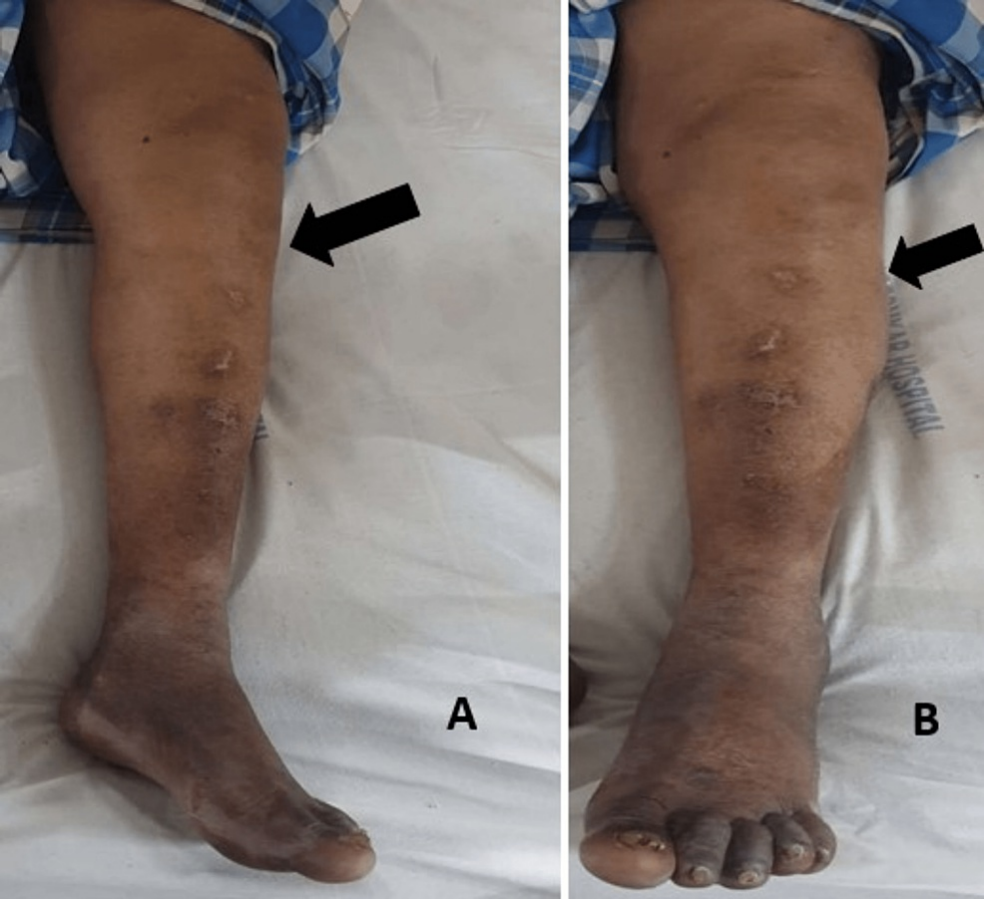
Clinical photograph of the left knee showing swelling A and B show clinical photographs of the swelling over the left proximal third aspect of the tibia

On clinical examination, the patient had tenderness and swelling on the anterolateral aspect of the left knee extending toward the proximal tibia (Figure 3). The swelling was well-defined, smooth, firm, and uniform in consistency with dimensions of 15 cm × 12 cm. Swelling was moving sideways suggesting that it is not attached to the bone but was adhered to the underlying soft tissue. Knee movements were painful and restricted. Paresthesia was not noted. General examination was normal.

**Figure 4:**
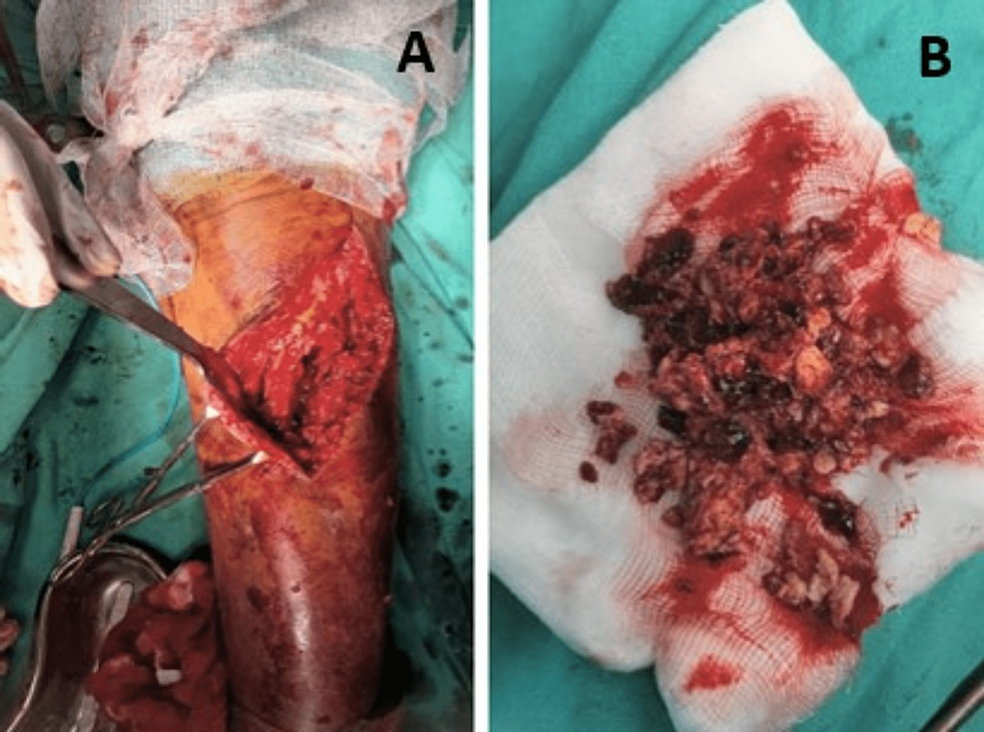
Intraoperative images of resected tumor mass A and B show intraoperative affected proximal tibia with a resected tumor mass

The patient was treated surgically under spinal anesthesia with curettage and bone grafting with bone cementing of the underlying tumor. A curvilinear incision was taken over the swelling on the anterolateral aspect of the knee. Soft tissue dissection was done. A cortical window was made in the bone. The tumor contains caesious material, which was removed with the help of a curette (Figure 4). Anteroposterior (AP) cortical breaches were noted in the bone. Repeated cycles of thorough curettage were done. At the end irrigation with normal saline given, the collected caseous material was sent further for histopathological examination.

**Figure 5:**
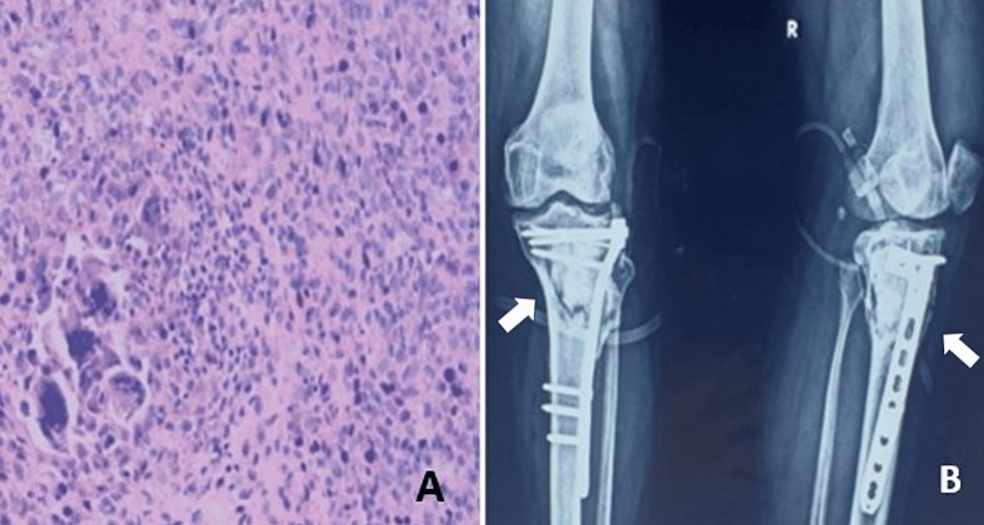
Histopathological image and immediate postoperative X-ray A and B show multinucleated giant cells on histological examination and anteroposterior and lateral X-rays after definitive surgery for the proximal tibia with plate osteosynthesis, bone cementing, and bone grafting

Bone graft was harvested from the inner cortical table of the same-side iliac crest. Harvested graft followed by gel foam was placed over the anteroposterior cortex breached areas of the bone and over the intercondylar region. The cavity was prepared for cementing (sandwich technique), and through the semisolid cement, a lateral locking proximal tibia plate was inserted. The excised tumor was sent for histopathological examination, which showed polygonal to round histiocytes surrounded by multinucleated giant cells, fibro-fatty tissue (Figure 5) suggestive of giant cell tumor of the bone.

Postoperatively, the physiotherapy treatment advised to the patient was paraffin wax bath for 20-30 minutes in three sessions to help relax the muscles and reduce pain and edema. To increase the range, some range of motion exercises were given, which include active assisted hip range of motion (ROM) exercises, active ankle ROM exercises, and passive knee ROM exercises. ROM exercises were given at intervals of 10 repetitions twice a day to prevent contractures and deformity.

## Discussion

The case reported in this article was presented to the hospital as a high-grade giant cell tumor of the left knee, which was diagnosed and confirmed on histopathological examination after the open biopsy. Excision of the tumor was surgically done with bone grafting and plating. At six weeks of follow-up, the patient does not show any clinical or radiological features of recurrence. The patient was asked to come for follow-up six weekly at least for a period of one year. Curettage was the mainstay of therapy for giant cell tumors, especially for grades 1 and 2, but associated with a high recurrence rate (35%-40%). Thus, adjuvants such as bone cement, phenol, hydrogen peroxide (H_2_O_2_), cryosurgery, and argon beam are used to reduce recurrence. Systemic therapies such as bisphosphonates, interferon alpha (IFN-a), and denosumab can be utilized to lower the likelihood of local recurrence. En bloc resection and reconstruction are the major treatments for grade 3. Other solutions for the reconstruction exist as well, such as cannulated cancellous (CC) screws and Steinmann pins; however, plating offers higher stability and rigidity.

In their study published in 2016 on Steinmann pin augmentation versus locking plate constructs, Ruskin et al. concluded that locking plate constructs had greater stiffness than tibial constructs fixed with Steinmann pins [12]. In their 2009 work, Uglialoro et al. came to the conclusion that locking plate constructs were stronger (P = 0.028) than Steinmann pin designs in a biomechanical investigation of distal femur defects treated using polymethylmethacrylate and internal stabilization devices [13]. Locking plate designs were substantially stronger than crossed-screw constructions (P = 0.001). Crossed-screw constructs failed due to defect bulging, articular impaction, and minimal fracture propagation; Steinmann pin constructs failed due to severe intra-articular fractures. Other groups have demonstrated that the use of locking plates is an effective treatment in other orthopedic oncology reconstructions. Locking plate systems, which work as fixed-angle devices, are believed to offer greater purchase in the bone of lower quality and similar buy with fewer screws, as well as to reduce screw pullout [14].

Postoperatively, follow-up was held every six weeks. The patient had complete relief of pain, with improvement in the range of movement, and knee flexion to 70 degrees with full extension without any surgical site complications evident. The patient was able to perform her activities of daily living. No evidence of recurrence was noted on clinical and radiological examination.

## Conclusions

Giant cell tumor of the bone in a left proximal tibia with a pathological fracture in an elder male was unusual. Thus, with the help of history and clinical examination with radiological and histopathological examination, we can reach a proper confirmatory diagnosis, which was helpful in proper preoperative planning, operative decision with complications, and possible outcomes.
